# Effect of grassland cutting frequency, species mixture, wilting and fermentation pattern of grass silages on in vitro methane yield

**DOI:** 10.1038/s41598-023-31964-3

**Published:** 2023-03-23

**Authors:** Kim Viggo Weiby, Sophie J. Krizsan, Ingjerd Dønnem, Liv Østrem, Margrete Eknæs, Håvard Steinshamn

**Affiliations:** 1 Faculty of Biosciences, Norwegian University of Life Sciences, 1432 Ås, Norway; 2 TINE SA, BTB-NMBU, PO Box 5003, 1432 Ås, Norway; 3 Department of Animal Nutrition and Management, Swedish University of Agricultural Sciences, 750 07 Uppsala, Sweden; 4 Division of Food Production and Society, Department of Grassland and Livestock, Norwegian Institute of Bioeconomy Research (NIBIO), 6967 Hellevik i Fjaler, Norway; 5 Division of Food Production and Society, Department of Grassland and Livestock, Norwegian Institute of Bioeconomy Research (NIBIO), 6630 Tingvoll, Norway

**Keywords:** Biochemistry, Biological models

## Abstract

Mitigating enteric methane (CH_4_) emissions is crucial as ruminants account for 5% of global greenhouse gas emissions. We hypothesised that less frequent harvesting, use of crops with lower WSC concentration, ensiling at low crop dry matter (DM) and extensive lactic acid fermentation would reduce in vitro CH_4_ production. Timothy (T), timothy + red clover mixture (T + RC) or perennial ryegrass (RG), cut either two or three times per season, was wilted to 22.5% or 37.5% DM and ensiled with or without formic acid-based additive. Silages were analysed for chemical composition and fermentation products*. *In vitro CH_4_ production was measured using an automated gas in vitro system. Methane production was, on average, 2.8 mL/g OM lower in the two-cut system than in the three-cut system (*P* < 0.001), and 1.9 mL/g OM lower in T than in RG (*P* < 0.001). Silage DM did not affect CH_4_ production (*P* = 0.235), but formic acid increased CH_4_ production by 1.2 mL/g OM compared to the untreated silage (*P* = 0.003). In conclusion, less frequent harvesting and extensive silage fermentation reduce in vitro CH_4_ production, while RG in comparison to T resulted in higher production of CH_4_.

## Introduction

Global warming caused by increased concentrations of greenhouse gases (GHGs) in the atmosphere is a major threat to the planet^1^. Food systems contribute up to 30% of global GHG emissions^2^, and methane (CH_4_) from ruminant production systems contributes to 5% of global GHG emissions^3,4^. Methane is 20 times as potent greenhouse gas as carbon dioxide (CO_2_), and its contributing share to global warming is increasing^5^. Enteric CH_4_ is produced by the removal of excess hydrogen (H_2_) and CO_2_, which results from the ruminal fermentation of feed carbohydrates, such as cell wall polymers, fructans, and starch, into volatile fatty acids (VFA)^6^. Therefore, finding a means to reduce enteric CH_4_ is crucial.

Forages such as grass and grass-clover silage (hereafter grass silage) constitute a large part of ruminant diets in Northern and Western Europe, as well as in Northern America. In Norway, ruminant production systems are located at approximately 58° to 71°N and within the coastal and alpine parts of the country. The growing degree-days, defined as accumulated mean temperature above 5 °C, is between 700 and 1200 °C, and the annual precipitation ranges from less than 300 mm to 4000 mm. Hence, climatic conditions for herbage production vary greatly^7^. Agricultural practices like cutting frequency, use of different species mixtures, wilting and use of silage additives also vary partly according to the climatic conditions.

In vitro studies have shown that advanced maturity of the forage used in the ensilage with decreased organic matter digestibility (OMD) at harvest resulted in a linear decrease in in vitro CH_4_ production per unit of feed dry matter (DM) incubated but increased CH4 out per g DM digested^8^. Purcell et al.^9^ found no difference between grass species in in vitro CH_4_ production (mL per gram of DM incubated). Genotypes of perennial ryegrass have been bred for high concentration of water-soluble carbohydrates (WSC) as measure to improve animal performance^10^, and such high sugar grasses are more prone to display extensive lactic acid fermentation during the ensiling process^11^. The readily available WSC in grass silage is subjected to fermentation, where lactic acid is the major end product in well-fermented silage. In the rumen, lactic acid is transformed into propionate^12^, and it is therefore possible that silages with high concentrations of lactic acid produce lower amounts of enteric CH_4_ compared to restrictively fermented silages. On the other hand, a recent study^13^ showed that increased concentrations of WSC in grass and grass-clover silage increase in vitro CH_4_ production possibly due to increased butyrate and acetate concentrations in the rumen fluid.

Navarro-Villa et al.^14^ found that perennial ryegrass had greater in vitro CH_4_ production (mL/g DM incubated) compared to red clover (*Trifolium pratense* L.) cultivars. Red clover contains less fibre (NDF) compared to perennial ryegrass^15^. Reduced NDF concentration might increase the propionate:acetate ratio in in vitro rumen fluid and reduce CH_4_ production, as feed with less fibre gives higher H_2_ concentration, more propionate and therefore less CH_4_ as propionate formation competes with methanogenesis for H_2_ in rumen^16,17^. Plant secondary compounds, such as condensed tannins or polyphenol oxidase, may have a specific lowering effect on enteric CH_4_ production^18,19^, but results are not unequivocal, as some in vivo studies show no effect of red clover on CH_4_ production^20^.

Wilting grass during silage production reduces water activity with immediate reduction in microbial activity and fermentation intensity during preservation^21^. Elevated DM concentration and reduced fermentation intensity in silage retain more WSC in the silage^22,23^. In addition, the use of formic acid-based additives that restrict fermentation can potentially preserve silage concentrations of WSC compared to silages prepared without additives or with the use of lactic acid bacteria inoculants^24,25^.

Although the above-cited studies indicate that forage species, harvest frequency, wilting and use of additives affect CH_4_ production, to the best of our knowledge no attempts have been made to compare combined effects of these factors. Therefore, the aim of this study was to test the effect of cutting frequency, growth period, crop type, wilting and fermentation pattern on in vitro CH_4_ production using a fully automated gas in vitro system. We hypothesised that (1) less frequent harvesting with longer growth periods, (2) use of ley species with lower WSC concentrations, (3) low crop DM and (4) extensive silage fermentation reduce in vitro CH_4_ production.

## Results

### Dry matter production, clover proportion and mean stage by count

Total annual DM yield was, on average, 7% greater in the two-cut system compared to the three-cut system (*P* < 0.001, Table 1). Timothy (T) obtained 16% less DM yield compared to perennial ryegrass (RG) across different harvest systems (*P* < 0.001), but 10% greater DM yield compared to the timothy red clover mixture (T + RC) (*P* < 0.001).

**Table 1:** Effect of Cut (1–5 where 1,3 and 5 is the first, second and third cut in the three-cut system, and 2 and 4 is the first and second cut in the two-cut system, respectively) and crop (T, timothy; T + RC, timothy + red clover; RG, perennial ryegrass) on dry matter yield per cut and total annual yield (n = 3).

	Harvest	Three cuts			Two cuts			SEM	*P* value							
		T	T + RC	RG	T	T + RC	RG		Cut	Crop	Cut × crop	C1	C2	C3	C4	C5
DM yield, g/m^2^	1st	465	458	602	729	740	837	21.72	< 0.001	< 0.001	0.023	< 0.001	< 0.001	0.215	< 0.001	< 0.001
	2nd	370	263	452	419	331	404									
	3rd	198	196	240												
	Total	1034	917	1294	1148	1071	1240	28.63	0.006	< 0.001	0.004	–	–	–	< 0.001	0.003

SEM is the standard error of the means. C1 is the contrast three versus two cuts per season, overall; C2 is the contrast three versus two cuts per season in the 1st cut; C3 is the contrast three versus two cuts per season in the 2nd cut; C4 is the contrast T versus RG; C5 is contrast T versus T + RC.

In the three-cut system, the first, second and third cuts accounted for 47%, 33% and 20% of the total annual DM yield across the different species mixture, respectively, while the first and second cuts accounted for 67% and 33% of the total annual DM yield, respectively, in the two-cut system.

The mean stage by count for T in the three-cut system was 2.69 and 2.72 in the first and second cuts, respectively, while it was 2.97 and 1.98 in the first and second cuts of the two-cut system, respectively. For RG, the mean stage by count in the three-cut system was 2.47 and 1.96 in the first and second cuts, respectively and 2.65 and 2.21 for the first and second cuts, respectively, in the two-cut system.

### Chemical characteristics of fresh and wilted materials

The concentration of WSC in fresh herbage was 33 g/kg DM greater in the three-cut system than in the two-cut system (124 vs. 91 g/kg DM respectively, *P* < 0.001, Table S1), while crude protein (CP) content was 27 g/kg DM greater in the three-cut system than in the two-cut system (132 vs. 105 g/kg DM respectively, *P* < 0.001). The NDFom concentration was 109 g/DM lower in the two-cut system than in the three-cut system (492 vs. 601 g/kg DM respectively, *P* < 0.001). The concentration of WSC was 56 g/kg DM greater in RG compared to T (162 vs. 106 g/kg DM respectively, *P* < 0.001) but there was no difference between T and T + RC (*P* = 0.306). The CP concentration was 10 g/kg DM lower in RG than in T (113 vs. 123 g/kg DM respectively, *P* = 0.01), but there was no effect of including red clover on the CP concentration of the fresh herbage (*P* = 0.264).

NDFom concentration was 60 g/kg DM lower in RG than in T (508 vs. 568 g/kg DM respectively, *P* < 0.001), and T + RC had 38 g/kg DM lower NDFom concentration than T (530 vs. 568 g/kg DM, *P* < 0.001). RG had a 22 g/kg DM lower NDFom than T + RC (508 vs. 530 g/kg DM, *P* = 0.006). In wilted herbage, concentrations of WSC were 13 g/DM greater in the two-cut system than in the three-cut system (119 vs. 106 g/kg DM respectively, *P* < 0.001, Table S2), while CP concentrations were 34 g/kg DM greater in the three-cut system than in the two-cut system (134 vs. 99.6 g/kg DM respectively, *P* < 0.001).

NDFom concentrations were 74 g/kg DM greater in the two-cut system than in the three-cut system (579 vs. 505 g/kg DM respectively, *P* < 0.001). Concentrations of WSC in wilted herbage were 36 g/kg DM greater in RG than in T (136 vs. 99.8 g/kg DM respectively, *P* < 0.001), but there was no difference between T and T + RC (*P* = 0.856). Concentrations of CP were 10 g/kg DM greater in T than in RG (123 vs. 113 g/kg DM respectively, *P* = 0.001), but there was no difference in CP concentrations between T and T + RC (*P* = 0.635). Concentrations of NDFom were 56 g/kg DM greater in T than in RG (566 vs. 510 g/kg DM respectively, *P* < 0.001), and T + RC was 37 g/kg DM lower in NDFom compared to T (566 vs. 529 g/kg DM respectively, *P* < 0.001).

Wilting had no effect on the concentration of ash, CP or NDFom (Table S3). The concentration of soluble CP as proportion of total CP increased with wilting. Wilting rate had an inconsistent effect on the concentration of WSC; it had no effect in T + RC and RG but increased the WSC concentration in T (Table S3).

### Effect of cut and crop types on silage chemical constituents and fermentation characteristics

The average NDFom concentration was 102 g/kg DM lower in the three-cut system compared to the two-cut system (451 vs. 553 g/kg DM respectively, *P* < 0.001, Table 2), and harvesting at a later stage of maturity resulted in 92 and 69 g/kg DM greater NDFom concentration in the first and second cut of the two-cut system compared to the three-cut system, respectively (*P* < 0.001).

**Table 2:** Effect of Cut (1–5 where 1,3 and 5 is the first, second and third cut in the three-cut system, and 2 and 4 is the first and second cut in the two-cut system) and crop (T, Timothy; T + RC, Timothy + red clover; RG, perennial ryegrass) on silage feed quality parameters averaged across DM and additive treatments (n = 4).

	Cut	Three cuts			Two cuts			SEM	*P* value							
		T	T + RC	RG	T	T + RC	RG		Cut	Crop	Cut × crop	C1	C2	C3	C4	C5
DM, g/kg	1st	31.1	31.9	29.3	35.2	37.3	34.8	4.666	0.481	0.918	0.999	0.200	0.198	0.916	0.724	0.994
	2nd	34.3	36.2	33.1	35.3	32.7	34.4									
	3rd	31.0	28.8	29.9	–	–	–									
Organic matter, g/kg DM	1st	930	932	924	942	945	932	0.762	< 0.001	< 0.001	< 0.001	< 0.001	< 0.001	< 0.001	< 0.001	< 0.001
	2nd	930	923	929	940	930	918									
	3rd	931	911	911	–	–	–									
NDFom, g/kg DM	1st	508	501	425	576	586	543	8.958	< 0.001	< 0.001	< 0.001	< 0.001	< 0.001	< 0.001	< 0.001	< 0.001
	2nd	487	429	487	562	515	535									
	3rd	449	370	400	–	–	–									
iNDF, g/kg NDFom	1st	119	112	121	230	220	246	6.268	< 0.001	< 0.001	< 0.001	< 0.001	< 0.001	0.011	< 0.001	0.518
	2nd	155	159	254	204	200	205									
	3rd	143	174	121	–	–	–									
OMD, %	1st	75.3	75.9	77.3	65.8	66.2	66.1	0.464	< 0.001	< 0.001	< 0.001	< 0.001	< 0.001	< 0.001	0.859	< 0.001
	2nd	73.7	75.3	67.9	68.1	70.1	69.0									
	3rd	75.5	76.3	77.9	–	–	–									
CP, g/kg DM	1st	147	126	121	103	89.3	95.4	1.709	< 0.001	< 0.001	< 0.001	< 0.001	< 0.001	< 0.001	< 0.001	0.008
	2nd	141	144	124	106	109	113									
	3rd	113	158	112	–	–	–									
sCP, g/kg CP	1st	790	755	781	580	557	721	24.85	< 0.001	< 0.001	0.012	0.002	< 0.001	0.181	< 0.001	0.002
	2nd	582	538	704	596	486	660									
	3rd	510	459	664	–	–	–									
WSC, g/kg DM	1st	37.6	46.4	91.5	60.4	58.0	83.8	21.78	0.639	0.160	0.641	0.961	0.619	0.649	0.218	0.502
	2nd	41.8	56.0	64.8	56.9	51.8	78.3									
	3rd	113	50.8	77.1	–	–	–									

DM, dry matter; NDFom, neutral detergent fibre; iNDF, indigestible NDF; OMD, organic matter digestibility; CP, crude protein; sCP, soluble crude protein; WSC, water-soluble carbohydrates. C1 is the contrast three versus two cuts per season overall; C2 is the contrast three versus two cuts per season, 1st cut; C3 is the contrast three versus two cuts per season, 2nd cut; C4 is the contrast T versus RG; C5 is contrast T versus T + RC.

The NDFom concentration was 38 g/kg DM lower in RG compared to T (478 vs. 516 g/kg DM respectively, *P* < 0.001), and 36 g/kg DM lower in T + RC compared to T (480 vs. 516 g/kg DM respectively, *P* < 0.001). The iNDF concentration was 66 g/kg NDFom lower across all cuts in the three-cut system compared to the two-cut system (151 vs. 217 g/kg NDFom respectively, *P* < 0.001).

Perennial ryegrass had 19 g/kg NDFom greater iNDF concentrations than T across all cuts (189 vs. 170 g/kg NDFom respectively, *P* < 0.001), but there was no significant difference between T and T + RC (170 vs. 173 g/kg NDFom respectively, *P* = 0.518). OMD was 7 percent point greater across all cuts in the three-cut system compared to the two-cut system (75 vs. 68%, respectively, *P* < 0.001). Postponing the first and second cuts resulted in 10 and 3 percent point lower OMD in the first and second cuts of the two-cut system compared to the three-cut system, respectively (*P* < 0.001).

There was no significant difference in OMD between T and RG (71.7 vs. 71.6%, respectively, *P* = 0.859), but 1.1 percent point greater OMD in T + RC compared to T (71.7% vs. 72.8%, respectively, *P* < 0.001). The concentration of WSC was not affected by either the harvest system (*P* = 0.961) or species mixture (*P* = 0.160). Silage concentration of lactic acid was, on average, 21 g/kg DM greater across all cuts in the three-cut system compared to the two-cut system (42 vs. 21 g/kg DM respectively, *P* = 0.001, Table 3). However, there was no significant difference between T and RG or between T and T + RC (*P* = 0.631 and *P* = 0.254, respectively).

**Table 3:** Effect of Cut (1–5 where 1, 3 and 5 is the first, second and third cut in the three-cut system, and 2 and 4 is the first and second cut in the two-cut system) and crop (T, Timothy; T + RC, Timothy + red clover; RG, perennial ryegrass) on silage fermentation characteristics averaged across DM and additive treatments (n = 4).

	Cut	Three cuts			Two cuts			SEM	*P* value							
		T	T + RC	RG	T	T + RC	RG		Cut	Crop	Cut × crop	C1	C2	C3	C4	C5
Lactic acid, g/kg DM	1st	35.6	33.3	38.8	22.6	18.9	23.3	11.31	0.007	0.515	0.889	0.001	0.129	0.104	0.631	0.254
	2nd	33.3	40.7	34.2	15.7	24.9	21.6									
	3rd	40.6	71.3	47.2	–	–	–									
Acetic acid, g/kg DM	1st	7.44	9.95	17.0	5.89	5.44	7.91	3.319	0.069	0.262	0.831	0.011	0.068	0.396	0.111	0.273
	2nd	8.09	9.38	9.90	5.71	7.77	6.92									
	3rd	10.3	16.6	12.7	–	–	–									
Propionic acid, g/kg DM	1st	2.35	1.93	1.99	1.00	0.88	1.47	0.199	< 0.001	0.251	0.392	< 0.001	< 0.001	0.037	0.583	0.104
	2nd	1.26	0.76	0.82	0.56	0.64	0.60									
	3rd	0.81	0.73	0.75	–	–	–									
Butyric acid, g/kg DM	1st	7.32	8.91	8.09	3.25	3.20	5.87	1.986	0.048	0.673	0.889	0.305	0.018	0.458	0.849	0.512
	2nd	3.37	2.58	3.89	6.03	4.19	3.25									
	3rd	5.76	2.71	5.84	–	–	–									
Ethanol, g/kg DM	1st	2.44	3.08	1.94	2.18	1.63	1.71	0.700	< 0.001	0.374	0.964	< 0.001	0.268	< 0.001	0.486	0.478
	2nd	1.28	1.74	1.29	5.27	5.11	4.51									
	3rd	1.46	2.67	1.64	–	–	–									
Formic acid, g/kg DM	1st	5.17	5.79	6.61	4.16	3.58	3.74	2.995	0.374	0.970	1.000	0.065	0.411	0.157	0.934	0.808
	2nd	5.72	5.35	5.01	1.47	2.69	1.37									
	3rd	5.57	6.99	6.15	–	–	–									
pH	1st	4.42	4.50	4.24	4.22	4.41	4.31	0.193	< 0.001	0.499	0.939	< 0.001	0.639	< 0.001	0.496	0.243
	2nd	4.04	4.21	4.06	4.80	4.98	5.21									
	3rd	4.08	4.19	4.16	–	–	–									
NH_3_, g/kg total N	1st	45.4	40.6	38.3	39.1	29.6	36.0	8.173	0.016	0.360	0.919	0.220	0.336	0.061	0.444	0.505
	2nd	33.1	31.9	46.8	56.9	50.4	64.3									
	3rd	38.9	43.6	47.9	–	–	–									

C1 is the contrast three versus two cuts per season overall; C2 is the contrast three versus two cuts per season, 1st cut; C3 is the contrast three versus two cuts per season, 2nd cut; C4 is the contrast T versus RG; C5 is contrast T versus T + RC.

Silage concentration of lactic and acetic acid was 20.5 (41.7 vs. 21.2 g/kg DM, respectively, *P* = 0.001) and 4.65 g/kg DM (11.3 vs. 6.61 g/kg DM respectively, *P* = 0.011) greater across all cuts in the three-cut system than in the two-cut system, respectively, but there was no difference between the different species (*P* = 0.515 for lactic acid and *P* = 0.262 for acetic acid). The concentration of propionic acid in the silage was 0.41 g/kg DM greater in the three-cut system than in the two-cut system (1.27 vs. 0.86 g/kg DM respectively, *P* < 0.001), but there was no difference between different species (*P* = 0.251). Concentrations of butyric acid and ammonia nitrogen (NH_3_-N) were not different between either harvest systems (*P* = 0.305 and *P* = 0.220, respectively) or species mixture (*P* = 0.673 and *P* = 0.360, respectively). Silage pH (5.0 vs 4.1, *P* < 0.001) and ethanol concentration (5.0 vs 1.4 g/kg DM, *P* < 0.001) were on average higher in the second cut of the two-cut than in the three-cut system, but did not differ between species mixtures (*P* = 0.499 and *P* = 0.374, respectively).

### Effect of wilting and the use of silage additives on silage chemical constituents and fermentation characteristics

The concentration of NDFom was 20 g/kg DM greater in silage made from herbage wilted to 37.5% DM compared to 22.5% DM (501 vs. 482 g/kg DM respectively, *P* = 0.040, Table 4), while there was no effect of silage additive on NDFom concentration (*P* = 0.398). Concentrations of ash, iNDF, OMD, CP or sCP were not affected by wilting level or silage additive. Concentrations of WSC were 25 g/kg DM greater in silage made from wilted herbage compared to unwilted herbage (77 vs. 52 g/kg DM respectively, *P* = 0.002) and 54 g/kg DM greater in silage preserved with additive than without (91 vs. 38 g/kg DM, *P* < 0.001, Table 4).

**Table 4:** Effect of wilting rate (target 22.5 or 37.5% DM) and silage additive (without or with) on silage feed quality parameters averaged across cuts and crop types (n = 15).

	22.5% DM		37.5% DM		SEM	*P* value						
	Without	With	Without	With		Cut	DM	Cut × DM	Add	Cut × Add	DM × Add	Cut × DM × Add
DM, g/kg	250	253	405	414	4.952	< 0.001	< 0.001	< 0.001	0.240	0.807	0.541	0.980
Organic matter, g/kg DM	929	928	928	928	2.126	< 0.001	0.778	0.994	0.834	0.997	0.780	0.999
NDFom, g/kg DM	481	483	510	493	9.187	< 0.001	0.040	0.976	0.398	0.967	0.313	0.789
iNDF, g/kg NDFom	179	183	179	170	7.767	< 0.001	0.434	0.813	0.731	0.998	0.411	0.952
OMD, %	72.3	71.9	71.3	72.4	0.526	< 0.001	0.623	0.975	0.452	0.995	0.183	0.707
CP, g/kg DM	119	119	121	121	3.816	< 0.001	0.606	0.995	0.946	0.930	0.975	0.999
sCP, g/kg CP	647	609	623	624	22.24	< 0.001	0.859	0.655	0.405	0.195	0.389	0.731
WSC, g/kg DM	18.7	85.7	56.8	97.0	7.513	0.243	0.002	0.505	< 0.001	0.381	0.082	0.050

DM, dry matter; NDFom, neutral detergent fibre; iNDF, indigestible NDF; OMD, organic matter digestibility; CP, crude protein; sCP, soluble crude protein; WSC, water-soluble carbohydrates. Cuts are numbered 1, 3 and 5 for the three consecutive cuts in the three-cut system and 2 and 4 for 1st and 2nd cut in the two-cut system, respectively. Cut × DM: Interaction between cut and dry matter; Add, Silage additive (with or without); Cut × Add: Interaction between cut and Add; DM × Add, interaction between DM and Add; Cut × DM × Add, three-way interaction between Cut, DM and Add.

The effect of additives on silage WSC content tended to be stronger at low compared to high wilting levels (DM by additive interaction, *P* = 0.082). Concentrations of lactic acid were, on average, 26 g/kg DM lower in silage made from herbage wilted to 37.5% DM compared to 22.5% DM (20 vs. 47 g/kg DM respectively, *P* < 0.001, Table 5) and 22 g/kg DM lower in silage preserved with additive than without (23 vs. 44 g/kg DM respectively, *P* < 0.001, Table 5). The effect of additive on silage lactic acid content was stronger in silage made from less wilted herbage than more extendedly wilted herbage (DM × Add, *P* < 0.001) and depended on cut (three-way interaction *P* = 0.025).

**Table 5:** Effect of wilting rate (target 22.5% or 37.5% DM) and silage additive (without or with) on silage fermentation characteristics averaged across cuts and crop types (n = 15).

	22.5% DM		37.5% DM		SEM	*P* value						
	Without	With	Without	With		Cut	DM	Cut × DM	Add	Cut × Add	DM × Add	Cut × DM × Add
Lactic acid, g/kg DM	61.7	31.4	26.9	13.9	2.475	< 0.001	< 0.001	0.183	< 0.001	0.003	0.001	0.025
Acetic acid, g/kg DM	17.7	7.83	8.70	3.41	0.861	< 0.001	< 0.001	0.493	< 0.001	0.022	0.012	0.290
Propionic acid, g/kg DM	1.54	0.98	0.99	0.91	0.079	< 0.001	< 0.001	0.619	< 0.001	0.094	0.005	0.637
Butyric acid, g/kg DM	8.50	5.64	3.95	1.71	0.705	< 0.01	< 0.001	0.345	< 0.001	0.140	0.662	0.534
Ethanol, g/kg DM	3.44	2.61	2.70	1.36	0.254	< 0.001	< 0.001	0.433	< 0.001	0.003	0.319	0.720
Formic acid, g/kg DM	0.70	12.3	0.28	5.22	0.377	< 0.001	< 0.001	0.505	< 0.001	< 0.001	< 0.001	0.821
pH	4.17	4.14	4.51	4.73	0.050	< 0.001	< 0.001	< 0.001	0.050	0.312	0.021	0.002
NH_3_, g/kg total N	63.4	41.3	38.0	28.8	2.102	< 0.001	< 0.001	0.020	< 0.001	0.877	0.004	0.854

Cuts are numbered 1, 3 and 5 for the three consecutive cuts in the three-cut system and 2 and 4 for 1st and 2nd cut in the two-cut system, respectively. Cut × DM: Interaction between cut and dry matter; Add, Silage additive (with or without), Cut × Add: Interaction between cut and Add, DM × Add: interaction between DM and Add, Cut × DM × Add, three-way interaction between Cut, DM and Add.

The effect of additive on silage lactic acid concentration was relatively stronger in the second and third cuts than in the first cut of the less wilted herbage of the three-cut system. Silage acetic acid concentrations were on average 6.7 g/DM greater in the silage wilted to 22.5% DM than in the silage wilted to 37.5% (13 vs. 6 g/kg DM respectively, *P* < 0.001), and 7 g/kg DM greater in silage preserved without silage additive than with additive (13 vs. 6 g/kg DM respectively, *P* < 0.001).

Concentrations of butyric acid in silage were, on average, 4 g/kg DM greater in low DM silages than in silages wilted to 37.5% (7 vs. 3 g/kg DM respectively, *P* < 0.001), and 2 g/kg DM greater in silage without additive compared to silage with additive (6 vs. 4 g/kg DM respectively, *P* < 0.001). Concentrations of NH_3_-N were 19 g/kg DM greater in low DM silages compared to silages wilted to 37.5% DM (52 vs. 33 g/kg DM respectively, *P* < 0.001), and 16 g/kg DM greater in silages without additive compared to silages with additive (51 vs. 35 g/kg DM respectively, *P* < 0.001).

### Effect of cutting system and crop type on in vitro rumen fermentation characteristics, in vitro total gas and CH_4_ production, and fractional rate of gas production

The molar proportion of acetate in rumen fluid was, on average, 0.014 mmol/mmol greater in silage made from the two-cut system than the three-cut system (0.618 vs. 0.632 mmol/mmol respectively, *P* < 0.001, Table 6), but the molar proportion of propionate did not differ between harvest systems (*P* = 0.547). Consequently, the acetate:propionate ratio was on average greater in the rumen fluid incubated with silages from the two-cut than three-cut system (3.07 vs. 2.98, *P* = 0.034). The molar proportion of butyrate was 0.007 mmol/mmol greater in the three-cut system compared to the two-cut system (0.104 vs. 0.097 mmol/mmol respectively, *P* < 0.001).

**Table 6:** Effect of Cut (1–5 where 1, 3 and 5 is the first, second and third cut in the three-cut system, and 2 and 4 is the first and second cut in the two-cut system) and crop (T, Timothy; T + RC, Timothy + red clover; RG, perennial ryegrass) on ensiled herbage in vitro rumen fermentation characteristics (total volatile fatty acids (VFA) and acids as molar proportion of total VFA), in vitro total gas CH_4_ production, and fractional rate of gas production (n = 4).

	Cut	Three cuts			Two cuts			SEM	*P* value^1^							
		T	T + RC	RG	T	T + RC	RG		Cut	Crop	Cut × crop	C1	C2	C3	C4	C5
Total VFA, mmol/L	1st	146	145	145	135	131	132	2.4	< 0.001	0.346	0.030	< 0.001	< 0.001	0.219	0.421	0.500
	2nd	139	140	135	138	134	133									
	3rd	132	144	138												
Molar Proportions																
Acetate, mmol/mmol	1st	0.621	0.628	0.600	0.634	0.636	0.619	0.003	< 0.001	< 0.001	< 0.001	< 0.001	< 0.001	0.001	< 0.001	0.007
	2nd	0.625	0.628	0.614	0.630	0.636	0.635									
	3rd	0.617	0.625	0.606												
Propionate, mmol/mmol	1st	0.200	0.201	0.221	0.205	0.207	0.216	0.003	< 0.001	< 0.001	0.009	0.547	0.534	0.979	< 0.001	0.986
	2nd	0.202	0.199	0.215	0.205	0.204	0.206									
	3rd	0.207	0.209	0.223												
Butyrate, mmol/mmol	1st	0.100	0.100	0.111	0.098	0.095	0.100	0.001	< 0.001	< 0.001	< 0.001	< 0.001	< 0.001	< 0.001	0.015	< 0.001
	2nd	0.104	0.104	0.103	0.099	0.097	0.094									
	3rd	0.107	0.100	0.108												
Acetate:Propionate	1st	3.10	3.13	2.72	3.12	3.09	2.88	0.053	< 0.001	< 0.001	0.016	0.034	0.469	0.238	< 0.001	0.445
	2nd	3.10	3.16	2.87	3.10	3.14	3.09									
	3rd	2.99	3.01	2.73												
CH_4,_ mL/ g DM	1st	32.2	31.4	35.8	28.2	27.5	30.7	0.79	< 0.001	0.005	0.003	< 0.001	< 0.001	0.353	0.003	0.815
	2nd	30.1	31.2	30.2	29.8	30.4	29.1									
	3rd	30.3	30.7	31.6												
CH_4_, mL/g OM	1st	34.6	33.7	38.7	30.0	29.1	32.9	0.86	< 0.001	< 0.001	0.002	< 0.001	< 0.001	0.299	< 0.001	0.460
	2nd	32.4	33.8	32.5	31.7	32.7	31.7									
	3rd	32.6	33.7	34.7												
CH_4_, mL/g DOM	1st	46.0	44.4	50.1	45.4	44.0	49.7	1.17	0.004	< 0.001	0.017	0.309	0.711	0.309	< 0.001	0.827
	2nd	43.9	45.0	48.0	46.5	46.7	45.9									
	3rd	43.2	44.2	44.5												
Total gas, mL/g DM	1st	194	196	204	178	176	186	4.1	< 0.001	0.226	0.264	< 0.001	< 0.001	0.268	0.183	0.732
	2nd	183	176	180	175	178	174									
	3rd	190	190	190												
Total gas, mL/g OM	1st	208	210	221	189	186	200	4.4	< 0.001	0.032	0.219	< 0.001	< 0.001	0.217	0.014	0.751
	2nd	197	191	194	186	192	190									
	3rd	204	209	209												
Total gas, mL/g DOM	1st	277	277	285	285	281	301	5.9	< 0.001	0.002	0.007	0.047	0.087	0.462	0.008	0.365
	2nd	268	254	286	272	274	274									
	3rd	270	274	267												
CH_4_, ml/L of total gas	1st	165	159	177	162	157	166	4.8	0.019	0.261	0.082	0.924	0.270	0.920	0.129	0.855
	2nd	164	178	168	172	171	168									
	3rd	160	160	165												
Fractional rate of gas production, /h	1st	0.058	0.061	0.064	0.058	0.055	0.060	0.0012	< 0.001	0.065	< 0.001	< 0.001	0.012	0.003	0.035	0.058
	2nd	0.058	0.061	0.059	0.056	0.056	0.055									
	3rd	0.060	0.064	0.061												

C1 is the contrast three versus two cuts per season, overall; C2 is the contrast three versus two cuts per season, 1st cut; C3 is the contrast three versus two cuts per season, 2nd cut; C4 is the contrast T versus RG; C5 is contrast T versus T + RC. DM, dry matter; OM, organic matter, DOM, digestible organic matter.

The molar proportion of acetate was 0.01 mmol/mmol greater in rumen fluid where silage made from T was incubated compared to RG (0.625 vs. 0.615 mmol/mmol respectively, *P* < 0.001) and 0.05 mmol/mmol greater in rumen fluid from T + RC than T (0.631 vs 0.625 mmol/mmol, respectively, *P* = 0.008, Table 6). There was no difference in the molar proportion of propionate between T and T + RC (*P* = 0.986). However, the molar proportion of propionate was 0.012 mmol/mmol greater in rumen fluid with RG than with T (*P* < 0.001), and the acetate:propionate ratio was greater in rumen fluid with T than RG (3.08 vs 2.86, *P* < 0.001). The molar proportion of butyrate was 0.001 mmol greater in RG compared to T (*P* = 0.015). Timothy resulted in a 0.003 mmol greater butyrate proportion compared to T + RC (*P* < 0.001).

CH_4_ production was, on average, 2.2 ml/g DM and 2.8 mL/g OM greater in silage made from the three-cut systems compared to the two-cut systems (31.5 vs 29.3 mL/g DM and 34.1 vs. 31.3 mL/g OM respectively, *P* < 0.001, Table 6). The first cut taken at a later stage of maturity reduced CH_4_ production by 4.6 ml/g DM and 5 mL/g OM compared to an earlier stage of maturity (30.6 vs. 35.7 mL/g OM respectively, *P* < 0.001), but there was no difference between harvest systems in the second cut (*P* > 0.2). CH_4_ production expressed per g digestible organic matter (DOM) was not affected by cutting system (*P* > 0.1, Table 6).

CH_4_ production was, on average, 1.3 ml/g DM, 1.9 mL/g OM and 2.6 mL/g DOM greater in silage from RG than T (31.5 vs. 30.1 mL/g DM, 34.1 vs. 32.2 mL/g OM, and 47.6 vs. 45.0 mL/g DOM, respectively, *P* < 0.01), but there was no difference between T and T + RC (*P* > 0.4).

Total gas production was, on average, 6 mL/g DM and 14 mL/g OM greater in silage from the three-cut system than the two-cut system (189 vs. 178 mL/g DM and 204 vs. 190 mL/g OM respectively, *P* < 0.001), but total gas produced per g DOM was on average greater in the two-cut system than in the three- cut systems (281 vs 273 ml/g DOM). Total gas production was 5 mL/g OM and 8 mL/g DOM greater in RG compared to T (202 vs. 197 mL/g OM and 283 vs 275 g/ DOM, respectively, *P* < 0.05). However, there was no difference between T and T + RC in total gas production (*P* > 0.3). There was no significant effect of cutting system or crop type on CH_4_ production relative to total gas production (Table 6).

The fractional rate of gas production was 0.004 units greater in the three-cut system compared to the two-cut system (0.061 vs. 0.057/h respectively, *P* < 0.001), and 0.002 units greater in RG compared to T (0.060 vs. 0.058/h, respectively, *P* = 0.035).

### Effect of wilting and use of silage additive on in vitro total gas and CH_4_ production, VFA production, and fractional rate of gas production

Rumen fluid molar proportion of acetate was 0.01 mmol/mmol greater from the incubation with wilted grass silage than the less wilted grass silage (0.628 vs. 0.618 mmol/mmol respectively, *P* < 0.001, Table 7), and silage preserved with additive also increased the molar proportion of acetate in the less wilted silage (*P* = 0.002). The molar proportion of propionate in rumen fluid was 0.01 mmol/mmol greater with the less wilted than with more extensively wilted grass silage (0.212 vs. 0.204 mmol/mmol respectively, *P* < 0.001), and the rumen fluid molar proportion of propionate was 0.01 mmol/mmol greater with grass silage preserved without additive than with additive (0.211 vs. 0.205 mmol/mmol respectively, *P* < 0.001). The actetate:propionate ratio in the rumen fluid increased both with wilting rate (2.93 vs. 3.10, *P* < 0.001) and with the use of additive (2.95 vs. 3.08, *P* < 0.001), but the effect of additive was stronger at 22.5% DM than at 37.5% DM as indicated by wilting rate by additive use interaction (*P* = 0.007, Table 7).

**Table 7:** Effect of wilting rate (target 22.5% or 37.5% DM) and silage additive (without or with) on silage in vitro rumen fermentation characteristics (total volatile fatty acids (VFA) and acids as molar proportion of total VFA), in vitro total gas and CH_4_ production, and coefficient of degradation (n = 15).

	22.5% DM		37.5%DM		SEM	*P* value						
	Without	With	Without	With		Cut	DM	Cut × DM	Add	Cut × Add	DM × Add	Cut × DM × Add
Total VFA, mmol/L	139	138	136	138	1.3	< 0.001	0.235	0.085	0.604	0.608	0.109	0.514
Molar Proportions												
Acetate, mmol/mmol	0.611	0.625	0.626	0.630	0.00	< 0.001	< 0.001	0.839	< 0.001	< 0.001	0.002	0.248
Propionate. mmol/mmol	0.217	0.207	0.205	0.202	0.001	< 0.001	< 0.001	0.923	< 0.001	0.023	0.027	0.400
Butyrate, mmol/mmol	0.103	0.102	0.100	0.100	0.001	< 0.001	0.002	0.687	0.490	0.665	0.512	0.381
Acetate:Propionate	2.83	3.03	3.06	3.13	0.028	< 0.001	< 0.001	0.850	< 0.001	0.007	0.019	0.447
CH_4_, ml/g DM	30.3	31.5	29.8	30.9	0.47	< 0.001	0.214	0.694	0.002	0.273	0.852	0.041
CH_4_, mL/g OM	32.6	34.0	32.1	33.3	1.11	< 0.001	0.235	0.721	0.003	0.272	0.817	0.056
CH_4_, mL/g DOM	45.1	47.2	45.1	46.0	0.72	0.005	0.318	0.493	0.007	0.455	0.266	0.183
Total gas, mL/g DM	186	188	181	184	2.6	< 0.001	0.019	0.333	0.130	0.155	0.978	0.060
Total gas, mL/g OM	200	203	195	198	2.9	< 0.001	0.025	0.359	0.143	0.185	0.931	0.076
Total gas, mL/g DOM	276	282	273	273	4.1	< 0.001	0.052	0.282	0.316	0.230	0.255	0.282
CH_4_, ml/L of total gas	163	169	164	169	2.7	0.031	0.750	0.362	0.033	0.374	0.838	0.774
Fractional rate of gas production, /h	0.060	0.060	0.057	0.060	0.0006	< 0.001	0.005	0.390	0.002	< 0.001	0.120	0.003

Cuts are numbered 1, 3 and 5 for the three consecutive cuts in the three-cut system and 2 and 4 for 1st and 2nd cuts in the two-cut system, respectively. Cut × DM: Interaction between cut and dry matter; Add, Silage additive (with or without); Cut × Add: Interaction between cut and Add; DM × Add, interaction between DM and Add; Cut × DM × Add, three-way interaction between Cut, DM and Add. DM, dry matter; OM, organic matter, DOM, digestible organic matter.

**Figure 1:**
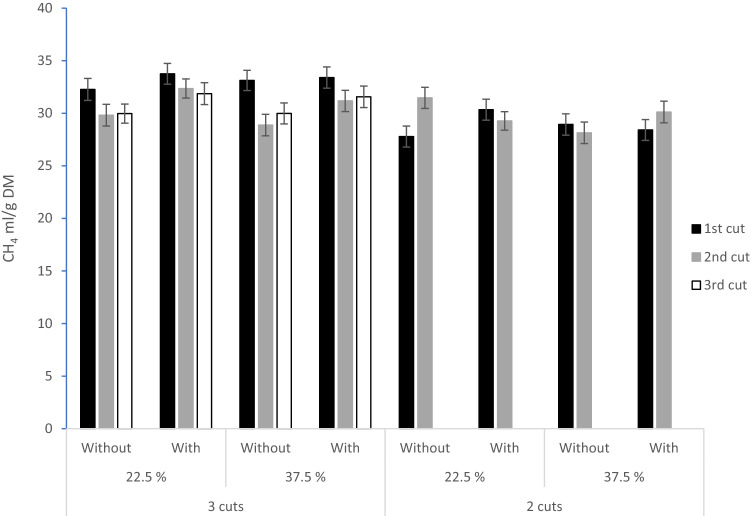
Three-way interaction between harvest regime (2 or 3 cuts per season), wilting levels (22.5% or 37.5% DM) and additive use (with or without formic acid-based additive) on in vitro methane production (mL/g DM). Bars represent standard error of the mean (n = 3).

There was no effect of herbage wilting rate on in vitro CH_4_ production (mL/g DM, mL/g OM, mL/g DOM, *P* > 0.2), but the use of silage additive increased in vitro CH_4_ production 1.2 mL/g DM, 1.2 mL/g OM, and 1.6 mL/g DOM compared to silage preserved without additive (31.2 vs 30.0 mL/g DM, 33.6 vs. 32.4 mL/g OM, and 46.6 vs. 45.0 mL/g DOM, respectively, *P* < 0.01). The effect of silage additive on CH_4_ production per g DM depended on cut and wilting rate, as indicated by a three-way interaction effect between cut, wilting rate and additive (*P* = 0.041). The interaction is illustrated in Fig. 1 showing that use of the additive generally increased CH_4_ production in the three-cut system but reduced CH_4_ production in the silage produced at the low wilting level of the second cut. Total gas production was not affected by using silage additive (*P* > 0.1), but the less wilted silage (22.5% DM) resulted in 4 mL/g DM and OM more gas production than the silage preserved at 37.5% DM (Table 7, *P* < 0.05). CH_4_ production of total gas production was 4.9 ml/L higher with the use of additive (168 vs 164 ml/L, *P* = 0.033, Table 7). Fractional rate of gas production was greater on low than high wilting rate (0.059 vs. 0.058/h respectively, *P* = 0.005) and greater with additive than without additive (0.060 vs. 0.058/h respectively, *P* = 0.002). However, the additive effect depended on both cutting system and wilting level, as indicated by the significant three-way interaction (*P* = 0.003). The use of additive increased the fractional rate of gas production in the three-cut system but decreased the rate in the second cut of the two-cut system (figures not shown).

### Correlations between grass silage parameters and in vitro CH_4_ production

The quantity of CH_4_ produced per g DM and OM incubated was positively associated with silage OMD (r = 0.47 and 0.53, respectively, *P* < 0.001, Table S4), formic acid concentration (r = 0.34 and 0.35, respectively, *P* < 0.01), and tended to be positively associated with silage WSC concentration (r = 0.21 and 0.22, respectively, *P* < 0.10). CH_4_ produced per g OM incubated was also positively associated with silage CP concentration (r = 0.28, *P* < 0.05) and CH_4_ produced per g DM tended to correlate with silage CP (r = 0.23, *P* < 0.10). Both CH_4_ produced per g DM and per g OM were negatively associated with silage NDFom concentration (r = − 0.45 and − 0.54, respectively, *P* < 0.001). The CH_4_ produced per g DOM incubated was negatively associated with silage OMD (r = − 0.31. *P* < 0.05), CP (r = − 0.26, *P* < 0.05), and lactic acid concentrations (r = − 0.35, *P* < 0.01), but tended to be positively associated with silage WSC concentration (r = 0.23, *P* < 0.10).

## Discussion

Higher total DM yield (Table 1) in the two-cut than in the three-cut system is in accordance with other studies^26,27^. Prolonged harvest interval with increased maturity of the plant in a two-cut system increases the proportion of cell wall structures with greater concentrations of aNDFom and total DM^28,29^.

Perennial ryegrass had a greater total DM yield than both T and T + RC. This was in accordance with a previous study investigating the difference between RG and T in harvest systems with four cuts per season, where T yielded more than RG only in the first cut^30^. Field trials of similar species and cultivars harvested annually two and three times showed similar DM yields for T and RG across two production years^31^.

The greater NDFom and iNDF concentration in the two-cut system compared to the three-cut system was a result of harvesting the crop at a more mature phenological stage in the two-cut system, with an increased proportion of cell walls and accumulation of indigestible lignin in the cell wall structures^28,29^. The effect of increased growth stage on increased silage concentration of NDFom, iNDF and reduced concentration of CP and reduced OMD, as seen in the present study, has also been reported in previous experiments on grass and clover silages^32,33^.

The lower fibre (aNDFom) concentration in RG compared to T in the present study has also been reported from a study in Ireland^34^. However, the greater iNDF concentration in RG, particularly of the second cut in the three-cut system, relative to both T and T + RC was surprising, especially since legumes like red clover normally have a greater iNDF:aNDFom ratio compared to grasses^15,35^. However, an increase in iNDF concentration of RG regrowth related to herbage mass has also been reported by Garry et al.^36^. The increased iNDF concentration of RG reduced OMD, which ultimately resulted in no difference between T and RG in OMD. The greater OMD in T + RC compared to T was expected, as the cell wall (NDF) concentration is normally lower in legumes compared to grasses and inhibition of cell wall (NDF) digestion by lignification with maturity is stronger in grasses than in legumes^37,38^.

The concentration of WSC was greatest and the concentration of lactic acid was lowest in silage wilted to 37.5% DM and treated with formic acid, which is in accordance with previous studies^24,39,40^. The addition of formic acid reduces pH by immediate acidification and restricts fermentation of WSC^39,41^, and wilting reduces microbial activity in the silage, thereby restricting fermentation intensity^21^.

The fermentation quality of the silages was in general more affected by the use of additives and wilting levels than harvest systems or species. Judged by the concentration of lactic acid, acetic acid, propionic acid, ethanol, NH_3_-N, and pH of the silages, the fermentation quality was acceptable, with generally low levels of fermentation products compared to other studies in grass and legume silage^42–44^. However, the butyric acid levels were high in silage with 22.5% DM and without additives (on average 8.5 g/kg DM, Table 5), which is not uncommon in silages without the use of chemical additives that prevent clostridia^45,46^. Higher concentration of ethanol in the second cut in the two-cut system (Table 3) than in the other cuts is difficult to explain. Usually, high concentrations of ethanol are associated with high numbers of yeasts^44^, which we did not record. However, even the highest ethanol concentrations observed in the current study are within levels regarded as typical (5—10 g/kg DM) for grass and legume silages^44^. The formation of NH_3_-N during ensiling is a result of degradation of plant protein caused by plant enzymes and proteolytic microbes like Clostrida. We have no records of the epiphytic flora or activity of plant proteases, but it is found that both plant proteases and epiphytic microbiota is affected by growth stage^47,48^. It has been reported higher protease activity and higher content of NH3-N in silage made from grass harvested at more mature growth stage than early^47^. The reduction of NH_3_-N concentration as a proportion of total-N in the silage with wilting and use of the formic acid-based additive is a consequence of restricted fermentation^42,44^. There were no association between silage ethanol and butyric acid concentration, NH_3_-N or ethanol concentration and in vitro CH_4_ production (Table S4).

The greater in vitro CH_4_ production observed for silage made from the three-cut system compared to the two-cut system coincides with greater OMD and lower aNDFom and iNDF concentrations. This is because CH_4_ is an end product from the rumen bacteria fermentation of digestible carbohydrates, like cell wall polymers and fructans, to VFA, H_2_ and CO_2_^49^. Holtshausen et al.^50^ also reported increased in vitro CH_4_ production (mL and mL/g NDF digested) when grass was ensiled from material harvested at early maturity, but no difference between grass maturity stages when CH_4_ production was expressed per g dry matter disappearance. We did not measure dry matter or organic matter disappearance in our in vitro cultures, but CH_4_ production per g digestible organic matter was not affected by maturity stage (Table 6) and as such in line with Holtshausen et al.^50^.

The acetate:propionate ratio in the rumen fluid was greater with silages made from the two-cut system than in the three-cut system, because of greater acetate production. It is well established that changes in ruminal VFA production towards a higher acetate:propionate ratio might increase CH_4_ production as acetate production generates H_2_ which is converted to CH_4_ by the methanogen microbiota^16^. However, in the present study, the greater acetate:propionate ratio in the two-cut system did not coincide with greater CH_4_ production. According to Johnson and Johnson^51^, there are two primary mechanisms regulating CH_4_ production, 1) the total amount of fermentable carbohydrates in the rumen and 2) changes in H_2_ supply through changes in VFA production. We speculate that the primary mechanism behind higher CH_4_ production in the three-cut silages in the present study was the amount of fermentable substrates, as indicated by greater OMD, giving higher total rumen fluid VFA production but only small effects on the molar proportions of actetate and propionate.

The on average greater in vitro CH_4_ production (mL CH_4_/g OM) from RG silages compared to that from T in the present study is in accordance with Purcell et al.^9^, who also observed greater in vitro CH_4_ production (mL/g DM incubated) in RG compared to T. Although the direction of rumen fluid fermentation in the present study was more methanogenic in T compared to RG, with a greater acetate:propionate ratio, the rate and extent of in vitro fermentation in RG was greater, as shown by the total gas (mL/g OM and DOM) and fractional rate of gas production (Table 6). This was likely a result of a greater total substrate availability, H_2_ production and CH_4_ production in RG compared to T. In addition, T had a greater NDFom concentration compared to RG.

A previous study showed that a greater NDFom concentration resulted in lower CH_4_ production^13^, which might explain why CH_4_ production was lower in T than RG in the present experiment. It has been shown that diets with red clover reduced in vivo CH_4_ production compared to diets with grass in cattle (17.8 vs 21.2 g/kg DM intake, respectively)^52^. However, in the present study, we did not observe lower CH_4_ production in T + RC compared to T. This supports earlier findings showing no such effect in diets with 60/40 perennial ryegrass and clover^20^. The inconsistency in the literature may be due to differences in forage quality, chemical composition, or herbage red clover proportion.

The silage produced without additive had a greater lactic acid concentration, resulting in a greater concentration of propionate when incubated in rumen fluid and was less methanogenic than silage produced with a formic acid-based additive. This is in line with a previous in vitro study that also demonstrated that propionic acid production in rumen fluid consumes H_2_ resulting in a lower in vitro CH_4_ output^53^. In vivo studies have also shown that lactic acid in silage is transformed into propionic acid in the rumen^54,55^. In addition, the silages produced with the additive contained residual formic acid (12.3 and 5.2 g/DM, Table 5), which may have contributed to higher CH_4_ production as demonstrated in other in vitro fermentation studies where increasing levels of formic acid or formate were added to the substrate^56,57^. This is supported by the positive association between CH_4_ production (ml/g DM and mL/g OM) and silage formic acid concentration in the correlation analysis of the present study (Table S4). Stronger effect of the formic acid-based additive on CH_4_ production in the three- than in the two-cut system and from the less wilted silage in the three-cut system (Fig. 1) is likely due to greater increase in rumen fluid acetate relative to propionate production (Table 7) and higher residual formic acid concentration (Table 5).

Previous studies have shown that increased DM concentration and reduced fermentation intensity in grass silage retain more WSC in the silage^22,23^. The present study also showed that DM levels affected WSC concentration. However, as we found no effect of wilting level and DM concentration on CH_4_ production, the role of WSC in affecting CH_4_ production was probably not as prominent as reported in other studies^11,13^. However, the correlation analysis indicated a tendency (*P* < 0.10) for a positive relationship between CH_4_ production and silage WSC concentration (Table S4).

This study showed that less frequent harvesting, extensive silage fermentation in the absence of silage additives, and the use of T as a grass species reduced in vitro CH_4_ production, while the use of formic acid based additive increased in vitro CH_4_ production. We recognise that these results must be confirmed in vivo along with animal production data.

In conclusion, our results confirmed the hypothesis that less frequent harvest and extensive silage fermentation reduce in vitro CH_4_ production. The effect of harvest frequency was mainly due to increased NDFom and iNDF concentration and reduced OMD in the two-cut system compared to the three-cut system, implying reduced amount of fermentable substrate available for rumen microorganisms. The effect of extensive silage fermentation was caused by an increased lactic acid production in the silage, increased rumen fluid propionate production and ultimately reduced CH_4_ production. Although we found that CH_4_ production was lower in T than in RG, this was probably not due to differences in WSC concentration but rather due to differences in the total substrate availability. Lastly, our results do not support the hypothesis that restricted lactic acid fermentation by wilting the crop before ensiling increases in vitro CH_4_ production, but that restricting silage fermentation by use of formic acid as an additive increase CH_4_ production most likely due to residual formic acid. Wilting resulted in a higher content of WSC, but there was no direct effect of DM level on in vitro CH_4_ production.

## Methods

### Experimental design

Silages were made from three crops: pure timothy (T; *Phleum pratense* L., cv. ‘Liljeros’), timothy and red clover mixture (T + RC; mixture of 85% timothy, cv. ‘Liljeros’ and 15% red clover, *Trifolium pratense* L., cv. ‘Gandalf’, based on seed weight) and pure perennial ryegrass (RG; *Lolium perenne* L., cv. ‘Figgjo’), harvested two (H2) or three (H1) times per season. After harvest, the crop was wilted to two different dry matter levels, target was 225 and 375 g DM/kg, and fermented with a formic acid-based additive, or without additive, and later analysed for chemical composition, fermentation products and in vitro and in situ characteristics. The design was factorial with two harvesting systems × three crops × two DM levels × two additive treatments. The field layout was a split plot design with a harvest regime on main plots and crop on sub-plots. There were four field replicates of all wilting rates and additive combinations within the harvests. Silages made from replicate 1–3 were used for further analysis, and silages made from replicate 4 were used as spare samples.

### Establishment of ley

The field trial was established on a medium sandy soil with high organic matter content (10.7% loss of ignition), pH of 5.9 and medium levels of plant available P (P-AL = 7.6 mg/100 dry soil) and K (K-AL = 7.9 mg/100 g dry soil). The crops were sown at a seeding rate of 25 kg ha^−1^ for T, 20 kg ha^−1^ timothy and 5 kg ha^−1^ clover in T + RC, and 35 kg ha^−1^ perennial ryegrass in RG, in four replicated blocks on 22 May 2019 at the Norwegian Institute of Bioeconomy Research, Fureneset (61°17.6′ N, 5°2.9′ E; elevation 30 m a.s.l.). Just before sowing, 4 hl ha^−1^ of lime, 35 tonnes ha^−1^ of cattle slurry + 60 kg N in NPK 18-3-15 was applied. In the spring of the first production year (year 2020), H1 plots received 150 kg N ha^−1^ in spring, 100 kg N ha^−1^ after the first cut, and 30 kg N ha^−1^ after the second cut, while the H2 plots received 160 kg N ha^−1^ in spring and 100 kg N ha^−1^ after the first cut. The clover plots (T + RC) received 50% of the N amount applied to the grass plots (T and RG). No weed control was needed.

The experiment was performed in accordance with all relevant institutional, national, and international guidelines and regulations for experimental research and field studies on plants/plant materials, such as the IUCN Policy Statement on Research Involving Species at Risk of Extinction and the Convention on the Trade in Endangered Species of Wild Fauna and Flora. The research did not involve rare or endangered species of fauna or flora, or species at risk of extinction. Timothy, red clover and perennial ryegrass are common species used in grassland cultivation in Norway; they are not protected species under national conservation laws and no permissions or licenses are required for the cultivation.

### Harvest

The crop was cut to a stubble height of 8 cm using a Haldrup grass harvester (J. Haldrup a/s, Løgstør, Denmark / Haldrup GmbH, Ilshofen, Germany). In the experimental year (2020), the first cut was taken on 2 June (H1) and 16 June (H2), the second on 14 July (H1) and 11 August (H2) and the third on 1 September (H1). The phenological development stage of timothy at harvest was determined as the mean stage by count^58^. At harvest, a grab sample was taken from each grass clover plot (T + RC) and frozen for later hand separation into clover and grass fractions. The samples were dried at 60° for 48 h and weighed. The red clover proportion (% DM yield) in the three-cut system of T + RC was 6%, 12% and 44% in the first, second and third cuts, respectively and 1% and 18% in the first and second cuts, respectively, of the two-cut system.

### Wilting and preservation

Approximately 2 kg of fresh crop from each plot were sampled at harvest and were frozen, while another 15 kg of fresh crop material was put into plastic mesh containers (6 containers with approximately 2.5 kg in each), weighed and moved indoors, where the crop from three boxes were force dried, in ambient temperature, to target level of 225 g DM/kg and the other three to target level of 375 g/kg DM. Target DM was verified by weighing the boxes regularly and final DM was determined by freeze drying. The wilted crop was chopped to lengths of 1–2 cm using a Hans-Ulrich Hege Saatzuchtmaschinen (Hohebuck, Waldenburg, Germany). From each of the two wilting levels, three chopped samples were taken and weighed to contain approximately 350 g DM each. One sample was frozen, while the two others were preserved as silage in evacuated and sealed polyethylene bags (Magic Vac IL VERO Scottvuoto, Flaem Nuova SpA., Brescia, Italy). Each bag was subjected to vacuum (− 1 bar) for about 60 s using a LAVA V300 Premium (Bad Saulgau, Germany). The control treatment (C) received no additive, while the other (G) received 4 ml/kg GrasAAT Lacto (Addcon Gmbh, Bitterfeld-Wolfen, Germany), containing 57%–67% formic acid, 14%–18% sodium formate, 1%–2% lactose. All bags were stored in a dark room with ambient temperature for 3 months. Thereafter, the bags were frozen at − 20 °C until further preparation, chemical analysis, and in vitro gas testing.

### Sample preparation and chemical analysis

The fresh (n = 45) and wilted samples (n = 90) were lyophilised and milled using a Tecator Cyclotec 1093 mill (Foss Tecator AB. Högans. Sweden), 1 mm mesh screen and split in two, where one subsample was analysed chemically at the Swedish University of Agricultural Sciences (SLU, Umeå). The DM concentration was determined by oven drying at 105 °C for 16 h, and ash concentration was determined by combustion of the dried samples at 500 °C for 4 h^59^. CP concentration was calculated from the nitrogen concentration (N × 6.25) measured by the Kjeldahl method^60^, using a 2520 digestor, Kjeltec 8400 analyser unit and 8460 sampler unit (all from Foss Analytical, Hillerød, Denmark). The ash-corrected neutral detergent fibre (NDFom)concentration was determined with the filter bag technique in an Ankom200 Fiber analyser (Ankom Technology Corp., Macedon, NY) using a heat stable α- amylase and sodium sulfite^61^. The analyses are presented in Tables S1–S3.

The other subsample of fresh or wilted samples was analysed at LabTek, Norwegian University of Life Sciences (NMBU) for DM (104 °C, ISO 6496), WSC according to Randby et al.^62^ and buffer soluble CP (sCP) according to Licitra et al.^63^. The analyses are presented in Tables S1–S3.

The frozen silages were split into two subsamples. One subsample was sent frozen to the Swedish University of Agricultural Sciences (SLU, Ultuna) and analysed for pH (Metrhom, Herisau, Switzerland), NH_3_-N^64^, lactic acid, acetic acid, propionic acid, 2,3-butandiol and ethanol using high-performance liquid chromatography^65^. The analyses are presented in Tables 3 and 5.

The other silage subsample was lyophilised and split into three subsamples. Two subsamples were milled using a Tecator Cyclotec 1093 mill (Foss Tecator AB. Högans. Sweden), a 1 mm mesh screen; one was stored frozen as a spare sample without any processing, while the second was merged with the two other field replicates of the same treatment and stored frozen in a sealed plastic bag. The merged samples were split into two samples; one was used in the in vitro gas test at SLU Umeå, and analysed for DM, ash, nitrogen, and NDFom as described above for fresh and wilted samples. The analyses are presented in Tables 2 and 4.

The other merged sample was analysed for DM (60 and 104 °C), WSC and sCP at LabTek (NMBU) as described above for fresh and wilted material, and presented in Tables 2 and 4. The third freeze-dried silage subsample was milled using a Tecator Cyclotec 1093 mill (Foss Tecator AB, Högans, Sweden), a 2 mm mesh screen, merged with the two other field replicates of the same treatment for determination of indigestible NDF (iNDF) at SLU, Umeå.

### In situ and in vitro measurements

All experimental procedures involving animals were approved by the Swedish Ethics Committee on Animal Research (Umeå, Sweden) and in accordance with Swedish laws and regulations regarding EU Directive 2010/63/EU on animal research. The iNDF concentration of the samples was determined as NDF after 288 h in situ rumen incubation, as described by Krizsan et al.^66^, using three ruminal cannulated lactating Nordic Red cows. The cows were fed for at least 14 days before in situ incubation a total mixed ration consisting of grass silage and a concentrate mixture (0.6:0.4 on a DM basis) ad libitum, which covered the animal’s energy and protein requirement. Samples of 2 g were weighed into polyester bags with 11 μm pores and a pore area equal to 5% of the total surface area (Sefar Petex 07–11/5-cloth, Sefar AG, Heiden, Switzerland). Organic matter digestibility (OMD, g/g) was calculated from concentrations (g/kg DM) of iNDF and NDFom according to Huhtanen et al.^12^:

$${\text{OMD}} = 0.882{-}0.00121 \times {\text{iNDF}}{-}0.00011 \times {\text{NDF}}$$

Data on OMD and iNDF is presented in Tables 2 and 4.

Rumen fluid for the in vitro gas trial was collected approximately 2 h after morning feeding from two fistulated Nordic Red cows fed the same diet as described for the in situ measurements. The rumen fluid was kept in 2 steel thermoses that had been prewarmed and flushed with CO_2_ to ensure an anaerobic environment. The pH value of the rumen fluid (mean 6.27, standard deviation 0.12) was recorded (744 pH Meter; Metrohm Ltd., Herisau, Switzerland) before it was filtered through four layers of cheesecloth into a measuring cylinder continuously flushed with CO_2_.

A total of 483 mL of rumen fluid was transferred through a funnel into another measuring cylinder containing 483 mL of buffer solution mixed with micro- and macro minerals, as described by Menke and Steingass^67^, at 39 °C under constant stirring and continuous flushing with CO_2_.

Feed samples were incubated in 60 mL of buffered rumen fluid and placed in a water bath at 39 °C, with continuous agitation for 48 h. The in vitro gas production experiment was conducted using a fully automated gas production technique described by Cone et al.^68^, in which the total gas volume was automatically recorded at 0.2-h intervals and corrected for normal atmospheric pressure (101.3 kPa).

Gas samples for in vitro CH_4_ determination were sampled every 2, 4, 8, 24, 32 and 48 h from each bottle using a gas tight syringe (Hamilton, Bonaduz, Switzerland). The concentration of CH_4_ was determined by injecting 0.2 mL of gas into a Star 3400 (CX series) gas chromatograph (Varian Chromatography, USA) equipped with a thermal conductivity detector (TCD)^69^. After 24 and 48 h of incubation, one ML of rumen fluid was collected from the bottles, mixed with 200 μl of 22 M formic acid and stored at − 18 °C until analysis.

The concentration of VFA in the rumen fluid was determined using high-performance liquid chromatography (HPLC), and the acids were separated using a packet ReproGel H column (Ammerbuch, Germany). They were further detected with an RI 2414 detector (Waters Assoc, USA). These procedures were repeated in a total of seven runs and all samples were incubated at least three times (n = 3 runs/silage). All runs included 36 bottles; in each run, 30 bottles contained silage samples, four bottles contained standard hay and two bottles contained blanks (i.e., bottles contained only 60 mL buffered rumen fluid). The 60 silage samples (in triplicates) were randomly allocated to the seven in vitro runs and the same sample was never incubated more than once within a run and never in the same bottle. The analyses are presented in Tables 6 and 7.

### Statistical analyses

The data analysis of the constituents in fresh, wilted and ensiled forages were derived from linear mixed-effects models using the procedure GLIMMIX in SAS (Version 9.4, SAS Institute Inc., Cary, NC, USA).

The constituents in fresh and wilted material constituents were modelled with cut (numbered chronological from 1 to 5, where 1, 3 and 5 is the first, second and third cut in the three cuts system and 2 and 4 is the first and second cut in the two cuts system), crop (T, T + RC or RG), wilting level (225 or 375 g/kg DM) and their interactions as fixed effects and field replicate (1–3) as random effects.

In order to test the effect of harvest system and species mixture on the constituents in ensiled material, cut (1–5), seed mixture (T, T + RC or RG) and their interactions were treated as fixed effects, and silage additive (without or with GrasAAT Lacto) and wilting level (225 or 375 g/kg DM) as random effects. The effects of silage additive and wilting were tested with cut (1–5), silage additive (without or with GrasAAT Lacto), wilting level (225 or 375 g/kg DM) and their interactions as fixed effects and species mixture as random effect. The data for total gas production (mL/g OM), CH_4_ production (mL/g OM), fractional rate of gas production (/h) and VFA (mmol/L in vitro fluid) were analysed using the same model as for silage constituents but included in addition the fixed effect of run (1–7) and the random effect of bottle (1–36).

The effect of the harvest system (two or three cuts per season) across seasons and within cuts and the separation of crops were tested using orthogonal contrasts. Tukey’s test was used for pairwise comparisons of means. Significance of effects were declared at *P* ≤ 0.05 and trends 0.05 < *P* ≤ 0.10.

Residual normality was assessed using plots = residual panel option in GLIMMIX, with no data showing deviation from normal distribution.

Pearson correlation coefficients were calculated to determine relationships between the individual silage’s chemical composition, fermentation parameters and CH_4_ production using the procedure CORR in SAS.

Significance of effects were declared at *P* ≤ 0.05 and trends 0.05 < *P* ≤ 0.10.

## Supplementary Information

Supplementary Information.

## Data Availability

The datasets used and analysed during the current study are available from the corresponding author on reasonable request.
