# Supplementary material for: Effect of grassland cutting frequency, species mixture, wilting and fermentation pattern of grass silages on in vitro methane yield
Source: Sci Rep. 2023 Mar 23;13:4806. doi: 10.1038/s41598-023-31964-3 (PMC10036558; doi:10.1038/s41598-023-31964-3)
Supplement: Supplementary file 1 — Supplementary Information. [file 41598_2023_31964_MOESM1_ESM.docx]

Supplementary:

|  |  |  | Three cuts | | |  | Two cuts | | |  | *P*-values | | | | | | | |
| --- | --- | --- | --- | --- | --- | --- | --- | --- | --- | --- | --- | --- | --- | --- | --- | --- | --- | --- |
| Analysis |  | Cut | T | T + RC | RG |  | T | T + RC | RG | SEM | Cut | Mix | Cut × mix | 1 | 2 | 3 | 4 | 5 |
| DM, g/kg | | 1 | 185 | 201 | 178 |  | 244 | 276 | 188 | 0.95 | < 0.001 | < 0.001 | 0.000 | <0.001 | <0.001 | <0.001 | <0.001 | 0.605 |
|  |  | 2 | 224 | 221 | 226 |  | 283 | 277 | 224 |  |  |  |  |  |  |  |  |  |
|  |  | 3 | 269 | 214 | 221 |  |  |  |  |  |  |  |  |  |  |  |  |  |
|  |  |  |  |  |  |  |  |  |  |  |  |  |  |  |  |  |  |  |
| Ash, g/kg DM | | 1 | 70.5 | 68.5 | 78.8 |  | 59.7 | 56.8 | 70.3 | 2.17 | <0.001 | <0.001 | <0.001 | <0.001 | <0.001 | 0.124 | <0.001 | <0.001 |
|  |  | 2 | 71.4 | 78.5 | 72.0 |  | 63.0 | 70.8 | 79.8 |  |  |  |  |  |  |  |  |  |
|  |  | 3 | 72.8 | 92.7 | 92.6 |  |  |  |  |  |  |  |  |  |  |  |  |  |
|  |  |  |  |  |  |  |  |  |  |  |  |  |  |  |  |  |  |  |
| WSC, g/kg DM | | 1 | 97 | 115 | 211 |  | 74 | 76 | 140 | 7.5 | <0.001 | <0.001 | <0.001 | <0.001 | <0.001 | 0.137 | <0.001 | 0.306 |
|  |  | 2 | 101 | 101 | 126 |  | 107 | 113 | 137 |  |  |  |  |  |  |  |  |  |
|  |  | 3 | 152 | 103 | 198 |  |  |  |  |  |  |  |  |  |  |  |  |  |
|  |  |  |  |  |  |  |  |  |  |  |  |  |  |  |  |  |  |  |
| CP, g/kg DM | | 1 | 144 | 124 | 124 |  | 107 | 91.8 | 96.7 | 5.7 | <0.001 | 0.002 | <0.001 | <0.001 | <0.001 | <0.001 | 0.010 | 0.264 |
|  |  | 2 | 142 | 148 | 122 |  | 109 | 114 | 111 |  |  |  |  |  |  |  |  |  |
|  |  | 3 | 115 | 160 | 113 |  |  |  |  |  |  |  |  |  |  |  |  |  |
|  |  |  |  |  |  |  |  |  |  |  |  |  |  |  |  |  |  |  |
| sCP, g/kg DM | | 1 | 45.9 | 37.9 | 50.3 |  | 36.1 | 28.5 | 37.4 | 2.33 | <0.001 | <0.001 | 0.002 | <0.001 | <0.001 | 0.007 | <0.001 | 0.871 |
|  |  | 2 | 37.8 | 37.7 | 46.0 |  | 30.6 | 31.7 | 42.7 |  |  |  |  |  |  |  |  |  |
|  |  | 3 | 38.5 | 52.0 | 48.7 |  |  |  |  |  |  |  |  |  |  |  |  |  |
|  |  |  |  |  |  |  |  |  |  |  |  |  |  |  |  |  |  |  |
| NDFom, g/kg DM | | 1 | 538 | 529 | 449 |  | 654 | 658 | 588 | 11.8 | <0.001 | <0.001 | <0.001 | <0.001 | <0.001 | <0.001 | <0.001 | <0.001 |
|  |  | 2 | 541 | 499 | 532 |  | 615 | 550 | 539 |  |  |  |  |  |  |  |  |  |
|  |  | 3 | 490 | 417 | 434 |  |  |  |  |  |  |  |  |  |  |  |  |  |

**Table S1** Effect of Cut (1-5 where 1, 3 and 5 is the first, second and third cut in the three-cut system, and 2 and 4 is the first and second cut in the two-cut system) and crop (T, Timothy; T + RC, Timothy + red clover; RG, perennial ryegrass) on fresh herbage quality parameters (n=3). DM, dry matter; NDFom, neutral detergent fibre; CP, crude protein; sCP, soluble crude protein; WSC, water-soluble carbohydrates. Contrasts 1: Three vs. two cuts per season, overall. 2: Three vs. two cuts per season, 1. Cut. 3: Three vs. two cuts per season, 2. Cut. 4: Timothy vs. ryegrass. 5: Timothy vs. grass clover.

|  |  |  | Three cuts | | |  | Two cuts | | |  | *P*-values | | | | | | | |
| --- | --- | --- | --- | --- | --- | --- | --- | --- | --- | --- | --- | --- | --- | --- | --- | --- | --- | --- |
| Analysis |  | Cut | T | T + RC | RG |  | T | T + RC | RG | SEM | Cut | Mix | Cut×mix | 1 | 2 | 3 | 4 | 5 |
| DM, g/kg | | 1 | 337 | 353 | 319 |  | 362 | 382 | 359 | 1.40 | <0.001 | 0.127 | 0.017 | <0.001 | 0.000 | 0.902 | 0.047 | 0.503 |
|  |  | 2 | 362 | 375 | 353 |  | 378 | 349 | 366 |  |  |  |  |  |  |  |  |  |
|  |  | 3 | 340 | 298 | 317 |  |  |  |  |  |  |  |  |  |  |  |  |  |
|  |  |  |  |  |  |  |  |  |  |  |  |  |  |  |  |  |  |  |
| Ash, g/kg DM | | 1 | 70.3 | 69.7 | 77.8 |  | 62.2 | 59.0 | 70.0 | 2.68 | <0.001 | <0.001 | <0.001 | <0.001 | <0.001 | 0.031 | <0.001 | <0.001 |
|  |  | 2 | 69.8 | 75.9 | 72.3 |  | 59.4 | 68.7 | 79.6 |  |  |  |  |  |  |  |  |  |
|  |  | 3 | 69.0 | 89.8 | 90.0 |  |  |  |  |  |  |  |  |  |  |  |  |  |
|  |  |  |  |  |  |  |  |  |  |  |  |  |  |  |  |  |  |  |
| WSC, g/kg DM | | 1 | 58.9 | 66.5 | 68.8 |  | 89.5 | 85.5 | 149 | 12.0 | <0.001 | <0.001 | <0.001 | 0.005 | <0.001 | <0.001 | <0.001 | 0.856 |
|  |  | 2 | 70.5 | 107 | 119 |  | 130 | 127 | 134 |  |  |  |  |  |  |  |  |  |
|  |  | 3 | 150 | 109 | 207 |  |  |  |  |  |  |  |  |  |  |  |  |  |
|  |  |  |  |  |  |  |  |  |  |  |  |  |  |  |  |  |  |  |
| CP, g/kg DM | | 1 | 155 | 130 | 126 |  | 104 | 87.2 | 93.4 | 6.10 | <0.001 | 0.000 | <0.001 | <0.001 | <0.001 | <0.001 | 0.001 | 0.635 |
|  |  | 2 | 141 | 142 | 128 |  | 100 | 104 | 109 |  |  |  |  |  |  |  |  |  |
|  |  | 3 | 117 | 159 | 111 |  |  |  |  |  |  |  |  |  |  |  |  |  |
|  |  |  |  |  |  |  |  |  |  |  |  |  |  |  |  |  |  |  |
| sCP, g/kg DM | | 1 | 376 | 377 | 466 |  | 429 | 395 | 502 | 11.2 | <0.001 | <0.001 | 0.010 | <0.001 | <0.001 | <0.001 | <0.001 | 0.494 |
|  |  | 2 | 335 | 348 | 439 |  | 392 | 373 | 500 |  |  |  |  |  |  |  |  |  |
|  |  | 3 | 325 | 345 | 447 |  |  |  |  |  |  |  |  |  |  |  |  |  |
|  |  |  |  |  |  |  |  |  |  |  |  |  |  |  |  |  |  |  |
| NDFom, g/kg DM | | 1 | 559 | 550 | 476 |  | 621 | 631 | 566 | 9.9 | <0.001 | <0.001 | <0.001 | <0.001 | <0.001 | <0.001 | <0.001 | <0.001 |
|  |  | 2 | 554 | 490 | 524 |  | 586 | 534 | 537 |  |  |  |  |  |  |  |  |  |
|  |  | 3 | 509 | 438 | 446 |  |  |  |  |  |  |  |  |  |  |  |  |  |

**Table S2** Effect of Cut (1-5 where 1, 3 and 5 is the first, second and third cut in the three-cut system, and 2 and 4 is the first and second cut in the two-cut system) and crop (T, Timothy; T + RC, Timothy + red clover; RG, perennial ryegrass) on wilted herbage quality parameters, average cross wilting levels (n=6). DM, dry matter; NDFom, neutral detergent fibre; CP, crude protein; sCP, soluble crude protein; WSC, water-soluble carbohydrates. Contrasts 1: Three vs. two cuts per season, overall. 2: Three vs. two cuts per season, 1. Cut. 3: Three vs. two cuts per season, 2. Cut. 4: Timothy vs. ryegrass. 5: Timothy vs. grass clover

| Mixture | Wilting | DM, g/kg | Ash, g/kg DM | CP, g/kg DM | sCP, g/kg CP | NDFom,g/kg DM | WSC, g/kg DM |
| --- | --- | --- | --- | --- | --- | --- | --- |
| Timothy | Fresh | 241^c^ | 68^c^ | 123^abc^ | 308^e^ | 568^a^ | 106^de^ |
|  | Wilted, 22.5% | 276^b^ | 66^c^ | 124^abc^ | 364^d^ | 563^a^ | 89^e^ |
|  | Wilted, 37.5% | 436^a^ | 66^c^ | 123^abc^ | 378^cd^ | 568^a^ | 111^cd^ |
|  |  |  |  |  |  |  |  |
| Timothy + Red clover | Fresh | 238^c^ | 74^b^ | 128^a^ | 295^e^ | 531^b^ | 101^de^ |
|  | Wilted, 22.5% | 268^b^ | 73^b^ | 124^abc^ | 365^d^ | 530^b^ | 105^de^ |
|  | Wilted, 37.5% | 434^a^ | 73^b^ | 125^ab^ | 371^d^ | 528^b^ | 92^de^ |
|  |  |  |  |  |  |  |  |
| Ryegrass | Fresh | 208^c^ | 79^a^ | 113^bc^ | 397^c^ | 508^c^ | 162^a^ |
|  | Wilted, 22.5% | 269^b^ | 77^a^ | 112^c^ | 457^b^ | 505^c^ | 131^bc^ |
|  | Wilted, 37.5% | 416^a^ | 79^a^ | 115^bc^ | 485^a^ | 515^c^ | 140^b^ |
|  |  |  |  |  |  |  |  |
| SEM |  | 0.6 | 1.2 | 2.7 | 4.8 | 4.7 | 4.8 |
|  |  |  |  |  |  |  |  |
| *Effects* | Cut | <.001 | <.001 | <.001 | <.001 | <.001 | <.001 |
|  | Wilting (W) | <.001 | 0.34 | 0.803 | <.001 | 0.51 | 0.001 |
|  | Cut×W | <.001 | 0.454 | 0.498 | <.001 | <.001 | <.001 |
|  | Mixture (Mix) | <.001 | <.001 | <.001 | <.001 | <.001 | <.001 |
|  | Cut×Mix | <0.01 | <.001 | <.001 | 0.002 | <.001 | <.001 |
|  | Mix×W | 0.070 | 0.785 | 0.926 | 0.122 | 0.757 | <0.001 |
|  | Cut×Mix×W | 0.017 | 0.999 | 0.993 | 0.014 | 0.842 | <.001 |

**Table S3**. Composition of fresh and wilted herbage (target 22.5 and 37.5% DM) at harvest from ley species timothy, timothy and red clover mixture and perennial ryegrass and the levels of significance, averaged across cuts (n=15).

| Parameters,  g/kg DM | *In vitro* CH_4_ production | | |  | Silage digestibility and chemical composition | | | |  | Silage fermentation products | | | | | |
| --- | --- | --- | --- | --- | --- | --- | --- | --- | --- | --- | --- | --- | --- | --- | --- |
|  | mL/g DM | mL/g OM | mL/g DOM |  | OMD | CP | NDFom | WSC |  | LA | AA | PA | BA | FA | Et |
| CH_4_, mL/g OM | 0.99*** |  |  |  |  |  |  |  |  |  |  |  |  |  |  |
| CH_4,_ mL/g DOM | 0.68*** | 0.64*** |  |  |  |  |  |  |  |  |  |  |  |  |  |
| OMD, % | 0.47*** | 0.53*** | -0.31* |  |  |  |  |  |  |  |  |  |  |  |  |
| CP | 0.23† | 0.28* | -0.26* |  | 0.64*** |  |  |  |  |  |  |  |  |  |  |
| NDFom | -0.45*** | -0.54*** | 0.14 |  | -0.83*** | -0.61*** |  |  |  |  |  |  |  |  |  |
| WSC | 0.21† | 0.22† | 0.23† |  | 0.03 | -0.17 | -0.07 |  |  |  |  |  |  |  |  |
| Lactic acid (LA) | -0.04 | 0.01 | -0.35** |  | 0.41** | 0.38** | -0.56*** | -0.50*** |  |  |  |  |  |  |  |
| Acetic acid (AA) | 0.19 | 0.17 | -0.14 |  | 0.37** | 0.23 | -0.49*** | -0.50*** |  | 0.90*** |  |  |  |  |  |
| Propionic acid (PA) | 0.24† | 0.21 | -0.02 |  | 0.27* | 0.21 | -0.03 | -0.30* |  | 0.30* | 0.35** |  |  |  |  |
| Butyric acid (BA) | 0.07 | 0.06 | -0.09 |  | 0.19 | -0.05 | -0.06 | -0.28* |  | 0.40** | 0.45*** | 0.56*** |  |  |  |
| Formic acid (FA) | 0.34** | 0.35** | 0.17 |  | 0.25† | 0.14 | -0.32* | 0.43*** |  | -0.12 | -0.25 | -0.07 | -0.06 |  |  |
| Ethanol (Et) | -0.10 | -0.10 | 0.01 |  | -0.15 | -0.14 | 0.20 | -0.42*** |  | 0.07 | 0.15 | -0.03 | 0.35** | -0.24† |  |
| NH3-N, g/kg N | -0.13 | -0.11 | -0.06 |  | -0.06 | -0.07 | -0.01 | -0.51*** |  | 0.52*** | 0.57*** | 0.15 | 0.52*** | -0.32* | 0.69*** |

**Table S4**. Pearson correlation between *in vitro* CH_4_ production and grass silage parameters. OM, organic matter; DOM, digestible organic matter; OMD, organic matter digestibility; CP, crude protein; aNDFom, neutral detergent fiber; WSC, water-soluble carbohydrates; †P<0.1, * P < 0.05, ** P < 0.01, *** P < 0.001.
